# Structure determination of an unstable macromolecular complex enabled by nanobody‐peptide bridging

**DOI:** 10.1002/pro.4432

**Published:** 2022-09-27

**Authors:** Josefine Lorentzen, Dennis Vestergaard Pedersen, Trine Amalie Fogh Gadeberg, Gregers Rom Andersen

**Affiliations:** ^1^ Department of Molecular Biology and Genetics, Section for Protein Science Aarhus Universitet Aarhus Denmark

**Keywords:** complement, convertase, innate immunity, macromolecular complexes, nanobody, structural biology

## Abstract

Structure determination of macromolecular complexes is challenging if subunits can dissociate during crystallization or preparation of electron microscopy grids. We present an approach where a labile complex is stabilized by linking subunits though introduction of a peptide tag in one subunit that is recognized by a nanobody tethered to a second subunit. This allowed crystal structure determination at 3.9 Å resolution of the highly non‐globular 320 kDa proconvertase formed by complement components C3b, factor B, and properdin. Whereas the binding mode of properdin to C3b is preserved, an internal rearrangement occurs in the zymogen factor B von Willebrand domain type A domain compared to the proconvertase not bound to properdin. The structure emphasizes the role of two noncanonical loops in thrombospondin repeats 5 and 6 of properdin in augmenting the activity of the C3 convertase. We suggest that linking of subunits through peptide specific tethered nanobodies represents a simple alternative to approaches like affinity maturation and chemical cross‐linking for the stabilization of large macromolecular complexes. Besides applications for structural biology, nanobody bridging may become a new tool for biochemical analysis of unstable macromolecular complexes and in vitro selection of highly specific binders for such complexes.

## Abstract 2

PDB Code(s): 7NOZ;

**Abbreviations**
BC2‐hFPNb1: fusion protein of the two nanobodies BC2 and hFPNb1
BC2T: peptide tag recognized by the BC2 nanobody
BC2T‐FB: BC2T tagged complement factor B
BLI: biolayer interferometry
C3: complement component 3
C3b: complement component 3 fragment b
Cryo‐EM: cryo‐electron microscopy
EM: electron microscopy
FB: complement factor B
FD: complement factor D
FP: complement factor properdin
FPc: two chain FP monomer containing all TSR repeats
FPΔ2,3: two chain FP monomer lacking TSR2 and TSR3
FPΔ3: two chain FP monomer lacking TSR3
IPTG: isopropyl β‐d‐1‐thiogalactopyranoside
*K*_D_
: dissociation constant for complex
*k*_off_
: rate constant for dissociation
*k*_on_
: rate constant for association
Nb: nanobody
SCIN: staphylococcal complement inhibitor
SEC: size exclusion chromatography
TEV: tobacco etch virus
TLS: Translation–Libration–Screw
TSR: thrombospondin repeat
vWA: von Willebrand factor A

## 1 INTRODUCTION

**FIGURE 1:**
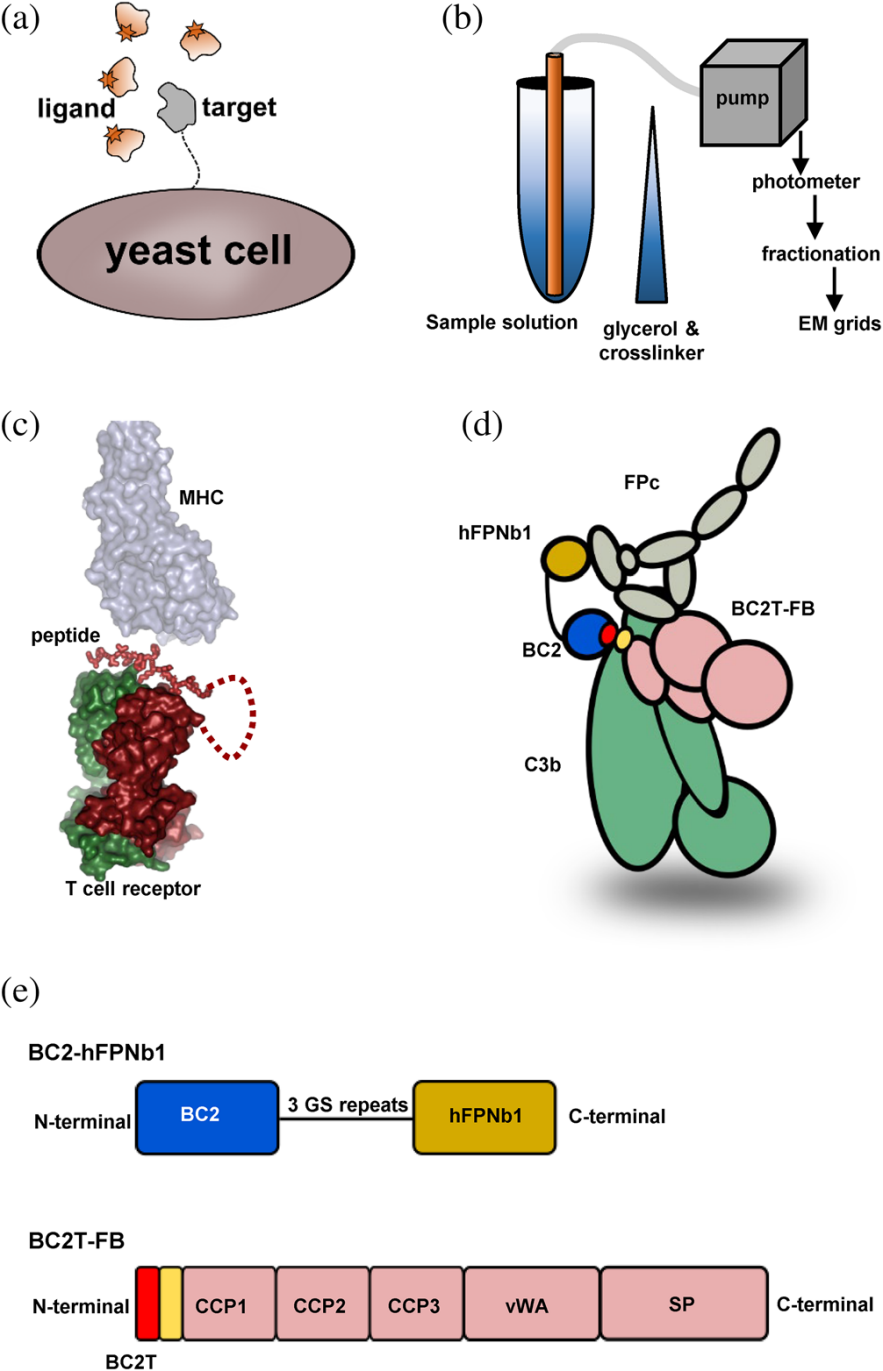
Strategies for stabilization of macromolecular complexes for structure determination. (a) Affinity maturation for a target subunit by display on, for example, a yeast cell. Target variants obtained by random mutagenesis are presented on the cells. The labeled binding partner is present in the fluid phase, and cells expressing target variants with improved affinity can be isolated by flow cytometry. (b) In gradient fixation, the labile complex is loaded on a preformed glycerol/cross‐linker gradient. After centrifugation, the cross‐linked complex is recovered and the relevant fraction used for preparation of electron microscopy (EM) grids. (c) A fusion protein containing at least two components of a labile complex increases the local concentration of interaction partners and thereby increases affinity. Shown here is the fusion of the MHC binding peptide to the N‐terminal end of the T‐cell receptor β‐chain through a flexible glycine rich linker. The peptide is optimally located for binding in the peptide binding groove of the MHC receptor prior to recognition of the MHC–peptide complex by the T‐cell receptor. Drawn from PDB entry 3PL6. (d) The BC2‐based strategy developed to tether factor properdin (FP) to the C3b:factor B (FB) complex. The BC2 nanobody recognizes the BC2 tag (red) inserted at the disordered N‐terminal end of FB (yellow, residues 26–34) and is fused to hFPNb1 that recognizes TSR4 within FP. (e) Schematic illustration of the bifunctional nanobody BC2‐hFPNb1 and BC2T‐FB

A major problem during structure determination is the dissociation of labile complexes during crystallization and preparation of grids for cryo‐electron microscopy (cryo‐EM). For 1:1 protein complexes, a dissociation constant (*K*_D_) below 10^−6^ M is preferable for both cryo‐EM and crystallography. Some well‐established strategies for stabilization of complexes are presented in Figure 1. Complex stability may be improved by affinity maturation of subunits, for example, interferon λ was matured allowing the structure of its complex with the receptor subunits IFN‐λR1 and IL‐10Rβ to be determined.^1^ Since the affinity matured subunit is not directly in vivo relevant, substantial validation of the resulting structure is required. It is also normally a requirement that the target subunit can be expressed on the surface of the cell (Figure 1a). For EM, chemical fixation where the labile complex is either sedimented through an increasing gradient of the cross‐linker (Figure 1b) or the cross‐linker is diffused into an agar matrix containing the labile complex has been used successfully for many targets.^2^, ^3^ However, cross‐linking is unpredictable and there is a risk of obtaining a mixture of conformations limiting the achievable resolution. Alternatively, complex stability can be increased by fusion of subunits through linkers to maintain a local high concentration. One example was provided by a (T‐cell receptor): (peptide–MHC) complex as outlined in Figure 1c, where the MHC binding peptide was fused to the N‐terminal end of the T‐cell receptor β‐chain.^4^ Whereas this works well for a peptide fused to a single protein, expression of large fusion proteins where multiple subunits are linked together may not be trivial. Tedious optimization of fusion geometry and linker length can be required to avoid formation of unspecific oligomers and the desired level of expression.

**FIGURE 2:**
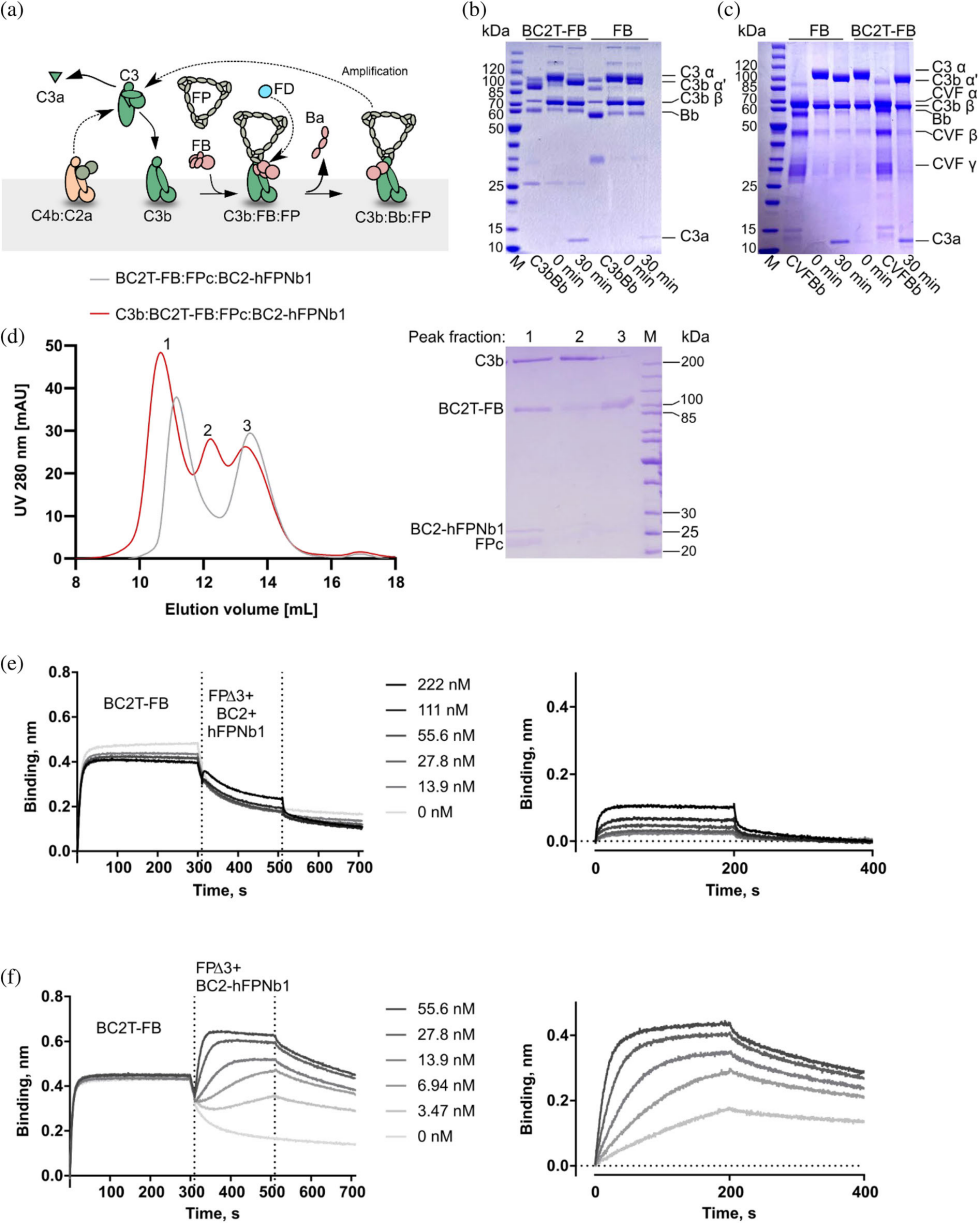
The alternative pathway of complement and biochemical characterization of the factor properdin–factor B (FP–FB) linking bifunctional nanobody. (a) The C3 convertase C4b:C2a is assembled upon activation of the upstream classical and lectin pathways. C3b generated by this convertase can associate with FB and FP to form the alternative pathway (AP) proconvertase complex C3b:FB:FP. Factor D cleavage converts this to the active AP C3 convertase C3b:Bb:FP, and since this convertase turns over multiple molecules of C3 before decaying, a strong amplification of the initial C3b deposition occurs. (b,c) C3 cleavage by the convertases C3b:Bb and CVF:Bb, panels (b) and (c), respectively, was monitored to compare the activity FB and BC2T‐FB. C3 cleavage to C3a and C3b was observed for both FB variants. (d) Size exclusion chromatography (SEC) analysis of the formation of the ternary complex BC2T‐FB:FPc:BC2‐hFPNb1 (gray chromatogram) and the quaternary complex C3b:BC2T‐FB:FPc:BC2‐hFPNb1 (red chromatogram). To the right, a nonreducing SDS‐PAGE analysis of the three peaks from the SEC analysis of the C3b:BC2T‐FB:FPc:BC2‐hFPNb1 complex (red chromatogram). An SDS‐PAGE analysis of the early peak of the ternary complex BC2T‐FB:FPc:BC2‐hFPNb1 (gray chromatogram) is presented in Figure S1b, (e) Left panel, biotinylated C3b was immobilized on a streptavidin coated biosensor and washed before binding to BC2T‐FB for 300 s. The biosensors were then washed for 10 s before exposed to a mixture of FPΔ3, BC2, and hFPNb1 in a 1:1:1 M ratio. Association and dissociation was monitored for 200 s. Right panel, association and dissociation phase after subtraction of the C3b:BC2T‐FB curve. (f) BLI experiment as in panel (e) but with the bispecific BC2‐hFPNb1 nanobody instead of the two separate nanobodies

The complement factor properdin (FP) enhances both recruitment of the zymogen factor B (FB) to surface bound C3b and the stability of the resulting C3b:FB complex known as the alternative pathway proconvertase^5^, ^6^, ^7^ (Figure 2a). In vivo, C3b is covalently bound to an activator of the complement cascade. Assembly of the C3b:FB complex leads to appearance of the alternative pathway convertase C3b:Bb upon activation of the C3b:FB proconvertase by factor D, deposition of additional C3b and subsequent strong amplification of the initial C3b deposition, reviewed in Reference 8. Comprehensive structural studies have elucidated the structures of the proconvertase C3b:FB and its complex with factor D,^9^ monomeric and oligomeric FP,^10^, ^11^, ^12^ and the active convertase C3b:Bb in the presence and absence of FP.^10^, ^13^ In contrast, our understanding of the mechanism whereby FP stimulates FB recruitment to C3b remains incomplete. We therefore aimed at structure determination of the C3b:FB:FP complex formed between complement C3b, the zymogen FB, and FP. By using an FP specific nanobody fused to a peptide specific nanobody, we stabilized the complex and determined its three‐dimensional structure with crystallography.

## 2 RESULTS

We have earlier determined the low‐resolution structure of the C3b:Bb:FP complex, which is the FD‐activated downstream version of the C3b:FB:FP target in the presence of the FP specific nanobody (Nb) hFPNb1.^7^, ^10^ Whereas we were able to isolate the C3b:FB:FP target complex by size exclusion chromatography (SEC), extensive crystallization screening was fruitless. Likewise, in 2D classes from negative stain EM, we observed extensive dissociation of the FP from the C3b:FB complex. We therefore investigated means of stabilizing the C3b:FB:FP complex. The downstream C3b:Bb:FP complex was stabilized by the bacterial protein SCIN for crystallization,^7^ but SCIN prevents formation of the upstream C3b:FB:FP target complex. By comparison with the structure of the C3b:FB complex,^9^ we prepared an in silico model of the C3b:FB:FP complex. Here, we noticed that if hFPNb1 bound to this complex, its N‐terminus would be approximately 60 Å from the FB Gly35 (prepro numbering). In 2016, the BC2 nanobody (Nb) specific for the 12 residue BC2T peptide tag was first described.^14^ Taking into account that FB residues 26–34 are disordered in known structures of C3b:FB, we predicted that FP and FB could be linked in a monodisperse stable complex if the BC2T was inserted N‐terminally of FB and the BC2 Nb was fused N‐terminally to the hFPNb1 Nb (Figure 1d). In bacteria, we expressed the fusion protein BC2‐hFPNb1 in which the C‐terminal end of BC2 was linked with a 15 residue linker (GGGGS)_3_ to the N‐terminal end of hFPNb1. In mammalian cell culture we expressed FB N‐terminally tagged with the BC2T peptide (Figure 1e and Table S1). The BCT2 tagged FB (BC2T‐FB) could be cleaved normally by complement factor D demonstrating that the BC2T inserted at the N‐terminal end of FB does not interfere with proconvertase assembly and FD activation (Figure S1a). In addition, we examined if BC2T‐FB could substitute for non‐tagged FB in C3 degradation when using either C3b or cobra venom factor (CVF) as the FB binding subunit. In these experiments, BC2T‐FB bound to either C3b or CVF followed by BC2T‐FB activation by FD, generating the convertase C3b:Bb or CVF:Bb. Next, the activated convertase was added to C3 to test, if BC2T‐FB could degrade C3 to C3a and C3b. From the assay, it is was concluded that BC2T‐FB can cleave C3 to C3a and C3b and thereby substitute for non‐tagged FB (Figure 2b,c).

Both natural and recombinant FP are oligomers of two to four subunits forming highly stable planar and extended structures that are unsuitable for high‐resolution structure determination.^11^ To allow structure determination, we therefore used our previously described two chain FP variants (FPc, FPΔ3, and FPΔ2,3) that are unable to oligomerize.^7^, ^15^ In FPΔ3 and FPΔ2,3, thrombospondin repeats (TSRs) 3 or 2 + 3 are deleted to obtain more compact FP molecules.^10^ All three FP variants interact with C3b and the C3b:FB complex and contain the binding site for hFPNb1 in TSR4.

To validate that we could assemble a monodisperse C3b:FB:FP complex stabilized by the bifunctional nanobody, we compared SEC runs for complexes where we used the two chain monomer FPc that contains all six thrombospondin repeats of FP.^7^ As expected, the BC2‐hFPNb1 fusion protein formed a ternary complex with BC2T‐FB and FPc (Figure 2d and Figure S1b). When C3b was added, a quaternary complex C3b:BC2T‐FB:FPc:BC2‐hFPNb1 eluted in a monodisperse peak consistent with a molecular weight of 350 kDa containing all four components in a stoichiometry of 1:1:1:1 (Figure 2d). Similar results were obtained with the smaller FP variants FPΔ3 and FPΔ2,3.

To evaluate whether linking of the two nanobodies increased the FP affinity for C3b:BC2T‐FB, we used biolayer interferometry (BLI). We compared the binding of FPΔ3 to a sensor bound to the preformed complex between immobilized biotinylated C3b and BC2T‐FB in the presence of the fusion BC2‐hFPNb1 or the two separate nanobodies. In line with prior studies, BC2T‐FB dissociation from C3b in the presence of the two non‐linked nanobodies was significantly slower than FPΔ3 association and FPΔ3 dissociation from the complex (Figure 2e). Subtraction of the C3b:FB curve from the C3b:FB:FPΔ3 curves in the presence of the two free nanobodies therefore provided a signal that mainly stems from FPΔ3 association but with a small contribution caused by slower dissociation of FB in the presence of increasing concentrations of FPΔ3 (Figure 2e and Figure S2a). In the presence of the BC2‐hFPNb1 fusion protein, an apparent faster association rate and a slower dissociation rate of FPΔ3 were observed as compared to the two separate nanobodies (Figure 2f and Figure S2b). Importantly, this was not due to monovalent binding of the BC2‐hFPNb1‐FPΔ3 complex binding to the BC2T tag on FB, since BC2T‐FB associated much more slowly to immobilized BCT2‐hFPNb1 (Figure S2c). Likewise, it was not due to monovalent association between the immobilized C3b and BC2‐hFPNb1‐FPΔ3 complex as the amplitude with the fused nanobodies was strongly increased compared to the experiment with separate nanobodies (compare panels (e) and (f) in Figure 2).

**FIGURE 3:**
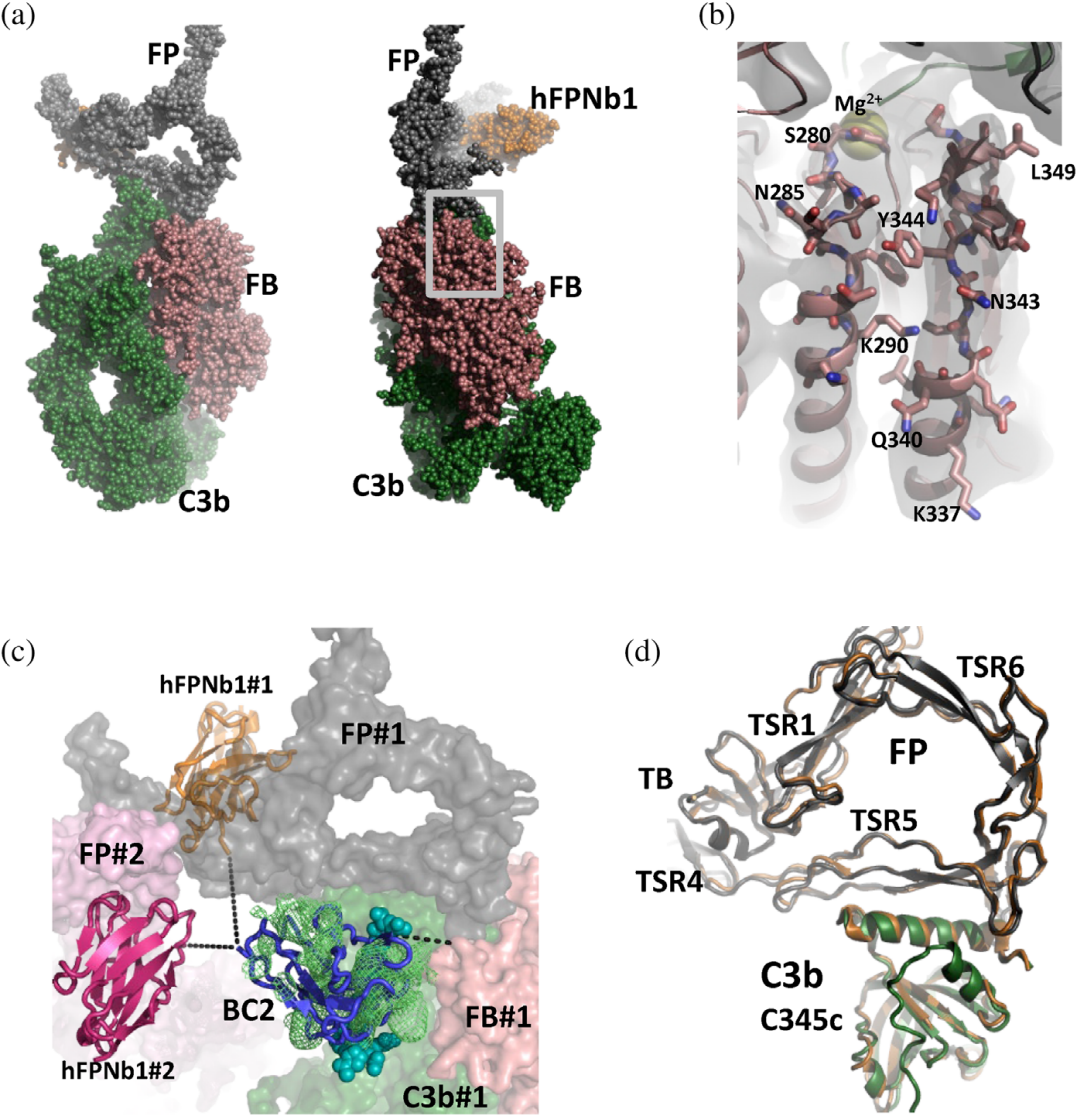
Nanobody bridging allows structure determination of the properdin (FP) bound proconvertase. (a) Crystal structure of the complex between the 175 kDa complement C3b (green), the 100 kDa BC2T‐factor B (FB) (salmon), the 46 kDa two chain FPΔ3 monomer (dark gray) and its associated 15 kDa hFPNb1 nanobody (orange). The overall dimensions of the complex are approximately 25 × 12 × 10 nm. C3b is covalently linked to the complement activating surface through a covalent bond. In the orientation shown here, the activator is at the bottom of the panel linked to the C3b thioester domain. (b) Expanded view of the region outlined in panel (a). In focus are two α‐helices in the FB von Willebrand factor A (vWA) domain that adopt different conformations in the C3b:FB:FP complex compared to the C3b:FB complex. A 2mF_o_‐DF_c_ electron density map contoured at 1.0 σ is displayed together with the model. (c) Docking of the BC2‐BC2T complex (nanobody in blue cartoon, peptide shown as cyan spheres) in residual electron density (light green) in a position compatible with fusion of the BC2T peptide to the N‐terminal end of FB. The C‐terminal end of the BC2 nanobody is closer to a symmetry related hFPNb1 (#2) compared to the hFPNb1 (#1) bound to the same complex as the nearby FB. (d) An overlay of FP (dark gray) and the C‐terminal domain of C3b (green) from the C3b:FB:FP complex and the C3b:Bb:FP complex (orange) demonstrates that the C3b‐FP interaction is preserved between the two functional states. For simplicity, labels FB and FP are used to represent BC2T‐FB and FPΔ3 in Figures 3 and 4.

To determine the structure of the C3b:BC2T‐FB:FPΔ3:BC2‐hFPNb1 complex, we reconstituted the complex and purified it by SEC and crystallized it (Figure S1c). Using diffraction data extending to 3.9 Å resolution, we determined the structure by molecular replacement. The final model (Figure 3a,b) obtained after iterative rebuilding and refinement has R/R_free_ values of 0.249/0.266 (Table 1). We also crystallized the C3b:BC2T‐FB:FPΔ2,3:BC2‐hFPNb1 complex and obtained diffraction data extending to 4.3 Å. The preliminary structure determined from these data is almost indistinguishable from the FPΔ3 containing complex and will not be described in detail.

**TABLE 1:** Data collection and refinement statistics

Data collection	
Resolution range	48.15–3.9 (4.039–3.9)
Space group	P 21 21 2
Unit cell	148.69 179.46 192.6 90 90 90
Unique reflections	47,648 (4688)
Multiplicity	39.7 (43.8)
Completeness (%)	99.80 (99.91)
Mean I/sigma (I)	10.85 (0.84)
R‐merge	0.1949 (6.295)
CC_1/2_	0.997 (0.399)
*Refinement*	
Reflections used in refinement	47,593 (4688)
Reflections used for R‐free	1849 (187)
R‐work	0.2488 (0.4651)
R‐free	0.2655 (0.4408)
Number of non‐hydrogen atoms	21,994
Macromolecules	21,631
Ligands	361
Solvent	2
Protein residues	2,754
RMS bonds (Å)	0.004
RMS angles (°)	0.73
Ramachandran favored/allowed/outliers (%)	94.85 4.71 0.44
Rotamer outliers (%)	0.33
Clashscore	2.43
Average B‐factor	288.9

*Note*: Statistics for the highest‐resolution shell are shown in parentheses. The table was prepared with the phenix.table_one program. The two solvent molecules are Mg^2+^ coordinating water molecules bound to the factor B MIDAS.

The hFPNb1 nanobody was bound to FPΔ3 TSR4 as expected and inspection of residual density within the crystal lattice guided us to dock a BC2:BC2T complex. In this putative location, the BC2 nanobody did not overlap with other molecules in the crystal lattice (Figure 3c). The C‐terminal end of the docked BC2T peptide was in reasonable distance of 21 Å from the first modeled residue in FB to which it is linked with nine residues. The closest alternative FB N‐terminal end was more than 90 Å away. Intriguingly, inspection of the packing revealed that the C‐terminal end of the docked BC2 nanobody was closer to the N‐terminal end of a symmetry related hFPNb1 (24 Å) compared to the hFPNb1 bound to the C3b:FB:FP complex to which the BC2T peptide was closest (39 Å). Considering the linker of 15 residues between the nanobodies, it is possible that the BC2 was linked to the symmetry‐related hFPNb1 (Figure 3c). If true, it appears that during association of a C3b:BC2T‐FB:FPΔ3:BC2‐hFPNb1 complex with a growing crystal or within the already formed crystal lattice, either of the two nanobodies may dissociate from its antigen and then reform the antigen interaction with a symmetry related antigen in the crystal lattice.

In our structure of the C3b:BC2T‐FB:FPΔ3 complex, FP binds to C3b essentially as in the downstream activated C3b:Bb:FP complex stabilized by the bacterial SCIN protein and in the core complex of the C‐terminal domain of C3b with FP.^10^, ^12^ Comparison of the C3b:FB:FP and the C3b:Bb:FP structures reveals that the C‐terminal domain of C3b and the FP TSR5 domain forming direct contacts superimpose with a root‐mean‐square‐deviation of 1.15 Å over 198 Cα atoms (Figure 3d).

**FIGURE 4:**
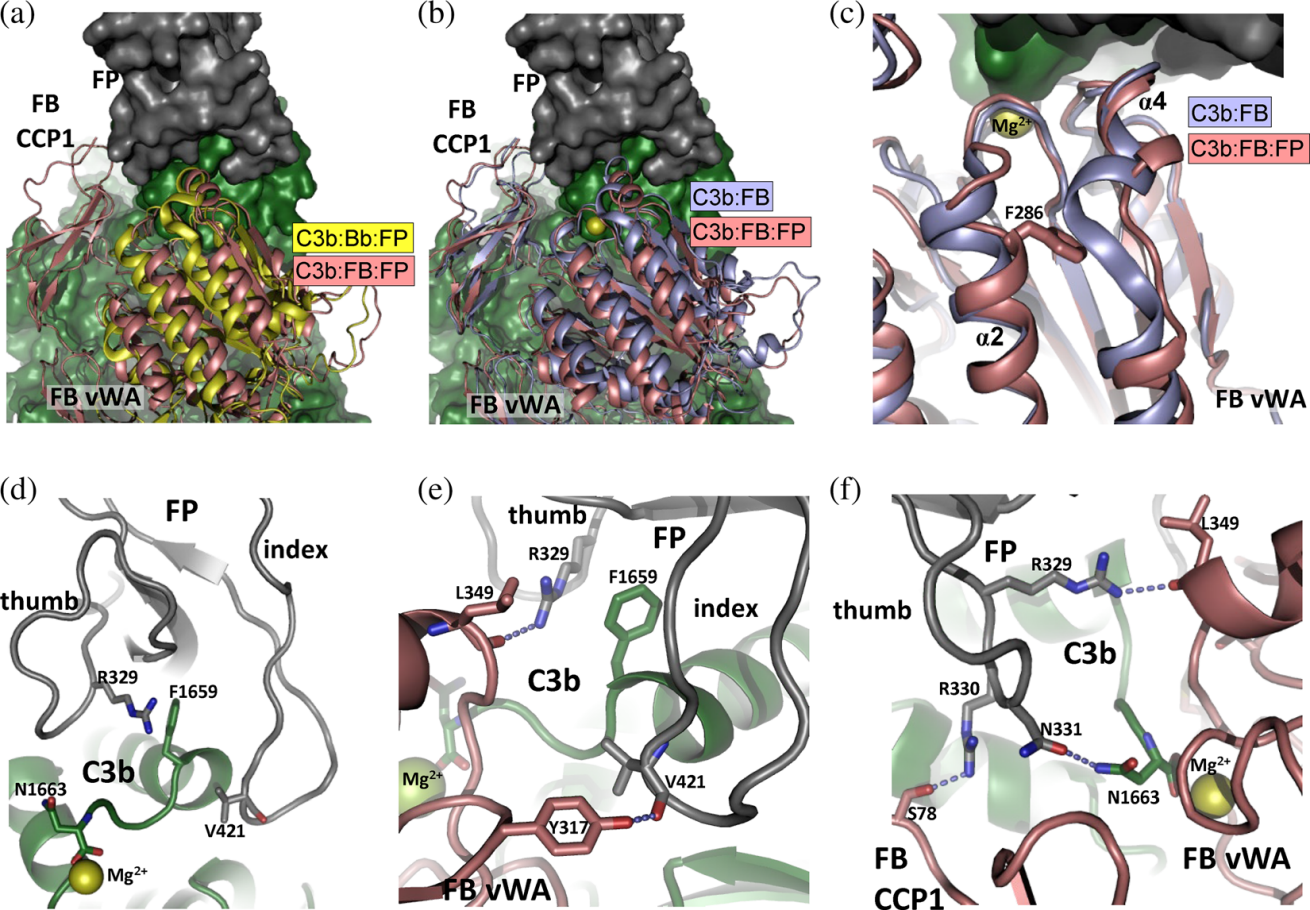
The factor B (FB) von Willebrand factor A (vWA) domain and the key role of properdin (FP) thumb and index finger loops. (a) Comparison of the vWA domain in the FP bound proconvertase (salmon) and convertase (yellow). Proconvertase cleavage by FD and release of the Ba fragment induces a rotation of the vWA domain. The structures are superimposed on the C‐terminal domain of C3b. (b) A comparison of the proconvertase in the presence and absence of FP demonstrates the rotation of the FB vWA domain induced by FP binding. The structures are superimposed on the C‐terminal domain of C3b. (c) Magnified view documenting the conformational change difference internally in the FB vWA domain between the two states of the proconvertase. The side chain of the Phe286 residue is shown in sticks, while the Mg^2+^ ion in the FB metal‐ion dependent adhesion site (MIDAS) is displayed as a yellow sphere. The structures are superimposed on their FB vWA domains. (d) From the C3b:FB:FP structure, the FP thumb and index finger loops and the C‐terminal region of C3b is displayed from a FB point of view. The Mg^2+^ ion from the FB MIDAS is indicated. (e) Close‐up of the intermolecular interface showing a direct interaction between the FB vWA domain and the FP index finger loop. (f) Likewise, the FP thumb loop forms polar interactions with both C3b and FB.

Turning to the C3b:FB interaction, the FB von Willebrand factor A (vWA) domain is overall rotated by 3° toward the FP TSR6 index finger loop in the C3b:FB:FP proconvertase when compared to the SCIN stabilized C3b:Bb:FP convertase (Figure 4a). Binding of FP vastly increases the recruitment of FB to C3b^5^, ^7^ and we therefore compared our structure of the FP stabilized proconvertase to those of the C3b:FB and C3b:FB:FD complexes. This revealed that FP binding induces significant changes in two α‐helices located in the vWA domain of FB close to the Mg^2+^ coordinating site (Figure 4b,c). Specifically, FB residues 344–349 in helix 4 and the N‐terminal end of the large α‐helix 2 shifts toward the FP index finger loop (Figure 4c). This shift is stabilized by an interaction apparently formed between the FP Arg329 side chain and the main chain oxygen of FB Leu349 (Figure 4e,f), both residues that are strictly conserved in mammalian FP and FB. This internal rearrangement in the FB vWA domain is accompanied by a 7° rotation of the domain relative to C3b as compared to the C3b:FB complex (Figure 4b,c). Mutation of FB Phe286 to leucine is known as a gain‐of‐function mutation and is associated with atypical hemolytic uremic syndrome where complement is dysregulated.^16^ This phenylalanine is central in the FB vWA domain residues that undergo conformational change upon binding of FP (Figure 4c). Our structure therefore possibly explains the gain‐of‐function phenotype.

PISA analysis^17^ of the C3b:FB:FP proconvertase complex reveals a small and polar FP–FB interface area of 273 Å^2^. The FP index finger loop in TSR6 and the FP thumb loop formed by residues 328–336 in TSR5 are involved in FP–FB interactions as also observed for the FP stabilized convertase C3b:Bb.^10^, ^12^ Within the C3b:FB:FP complex, the TSR5 thumb loop is located in a well‐defined pocket formed by the FB vWA domain, the first CCP domain in FB and the small α‐helix 1 in the C‐terminal domain of C3b (Figure 4f). In addition, FP–FB contacts are formed between the side chain hydroxyl of FB Tyr317 and main chain carbonyl of Val421 in the FP TSR6 index finger loop (Figure 4e). Also, Ser78 in the FB CCP1 domain engages in a hydrogen bond with FP Arg330 in the TSR5 thumb loop (Figure 4f), but since FB Ser78 and Tyr317 are not well conserved, these interactions may be less essential for FP stimulation of FB recruitment. Identification of water molecules bridging FP and FB is not feasible at this resolution, but bridging waters are likely to support the relatively few direct FP–FB interactions.

## 3 DISCUSSION

Due to their single domain nature, nanobodies are very easy to engineer and many examples of nanobody containing fusion proteins are known.^18^ Here, we fuse the BC2 nanobody to a second nanobody to accomplish tethering. Alternatively, the BC2 nanobody could be fused to any other kind of molecule. A third strategy could be to express a fusion protein with the BC2 nanobody linked to subunit A to accomplish proximity to the BC2T in subunit B. Our success relied heavily on substantial prior knowledge regarding the expected structure of the target complex and the known position of the FP specific nanobody hFPNb1. We predict that a more wide implementation of our strategy is enabled by alphafold2 for modeling of macromolecular complexes^19^, ^20^ combined with the ability to rapidly select many high‐affinity nanobodies by lama immunization^21^ or from synthetic libraries.^22^, ^23^ In addition, rapid mapping of nanobody location on macromolecules by negative stain EM^24^ may further direct the design of nanobody tethered macromolecular complexes suffering from weak affinity for one of the subunits.

One disadvantage of our strategy is that the BC2 moiety of the bispecific nanobody does not adopt a defined position relative to the remaining subunits of the stabilized complex and cannot contribute to crystal packing. In our case, the dimensions of the complex gives rise to large solvent channels that can accommodate the BC2 nanobody, but for smaller complexes crystallization could become difficult in the presence of a flexible BC2 linking nanobody. If the target complex is studied by cryo‐EM, a linking BC2 nanobody connecting subunits in a complex is likely to average out during 3D reconstruction. Another complication of our approach is that the N‐ and C‐terminals of the BC2T peptide are 27 Å apart. For this reason, insertion of BC2T inside a loop may require long linkers at either side of BC2T to allow binding to the BC2 nanobody. Taking advantage of alphafold2‐based models, sequence conservation analysis, and available prior experimental knowledge it appears feasible to identify loops in a target subunit in which the BC2T tag can be introduced without compromising folding of the tagged subunit. We foresee that flexibility of the inserted BC2T tag can be substantially increased, if a specific protease site is introduced at one of the ends of the inserted BC2T site and the purified tagged protein is cleaved specifically with the protease. By placing the protease site at either the N‐ or the C‐terminal end of the inserted BC2T site, one may vary the geometry. The geometry can be experimentally tested to find the most favorable for formation of monodisperse complex, ability to crystallize and generation of EM grids of a quality required for data collection.

Direct alternatives to the BC2 tag are the 13‐residue α‐tag and the four residue EPEA that also are peptide tags recognized by a nanobody.^25^, ^26^ Like the BC2 tag, these tags may also be introduced into subunits of labile macromolecular complexes. Both the BC2T and the α‐tag specific nanobodies recognize their antigen with low‐nanomolar affinity whereas the affinity of the NbSyn2 EPEA peptide is 240 nM.^25^ For tagging of internal loops, an UBC6e specific nanobody may also be convenient as demonstrated in a study where a nonessential loop in the parathyroid hormone receptor‐1 was replaced by the 14‐residue epitope for the UBC6e specific nanobody. Selective and increased receptor activation was obtained when the UBC6e nanobody fused in a C—C geometry to an agonist 14 residue fragment of the parathyroid hormone was used to activate cells presenting the parathyroid hormone receptor‐1.^27^

A different strategy for stabilization of unstable complexes for structural biology not taking advantage of nanobodies that resembles our approach was recently described. Here, a major luciferase fragment was fused to the C‐terminal end of the VIP1R G‐protein coupled receptor, while the complementary luciferase peptide was fused to the Gβ subunit of the heterotrimeric G‐protein.^28^ Upon peptide–luciferase interaction, the VIP1R‐Gαβγ complex was stabilized and significantly more monodisperse compared to the non‐stabilized complex and the structure of the complex could be determined by cryo‐EM single particle analysis. In terms of size, the two systems are comparable with 169 residues in the luciferase‐peptide complex compared to 135 residues in the BC2‐BC2T complex.

The structure of the FP stabilized proconvertase presented here offers a significantly improved foundation for understanding the FP mediated positive regulation of the complement alternative pathway. Comparison with prior structures of the FP bound C3b:Bb convertase^7^, ^10^ does not reveal major differences with respect to how FP interacts with C3b and FB/Bb. This argues that the presence of the *Staphylococcus aureus* protein SCIN used to trap an inactive dimer of the C3b:Bb convertase does not change significantly how FP interacts with C3b and Bb. Our structure of the FP bound proconvertase confirms the importance of the TSR5 thumb and the TSR6 index finger loops with respect to FP–FB interactions and as essential functional elements in FP. Accordingly, the FP‐targeting CirpA family of tick complement inhibitors tightly recognize the TSR5 thumb loop and cause disordering of the TSR6 index finger loop.^29^

Our structure also offers an opportunity to further investigate how polymorphisms in FP and FB can cause reduced activity of the complement cascade or gain‐of‐function that increases the activity. Reduced alternative pathway activity due to FP deficiency dramatically increases the risk of infection with especially *Neisseria meningitidis*.^30^ Excessive AP activity due to mutation in FB is also well described in relation to the atypical hemolytic uremic syndrome.^31^ In particular, the underlying reason for the enhanced in vitro recruitment of the FB variant Phe286Leu to C3b in the absence of FP^16^ may be rationalized by our structure of the C3b:FB‐FP complex. We speculate that the Phe286Leu mutation leads to an FB vWA domain conformation resembling that in the FP bound complex and thereby stimulates FB recruitment even in the absence of FP.

Besides structural biology and basic biochemical analysis, we foresee multiple other applications for nanobody bridged complexes. In relation to structural biology, the architecture of large macromolecular complexes is often studied with cross‐linking mass spectrometry as an orthogonal approach.^32^ Possibly, more unambiguous cross‐links may be obtained if the complex analyzed is stabilized through nanobody bridging during cross‐linking. Another obvious application is in vitro selection with immobilized nanobody bridged complexes favoring selection of binders that specifically recognize the complexes rather than stable subcomplexes or the free subunits. This could favor the development of highly specific modulators of large macromolecular complexes for therapeutic applications.

## 4 MATERIALS AND METHODS

### 4.1 Preparation of complement proteins

Human complement C3 was prepared in small scale essentially as described for rat C3.^33^ Alternatively, large‐scale preparation of C3, C3b, and biotinylated C3b was conducted as described in Reference 7. Recombinant FB and BC2T‐FB carrying the stabilizing mutation D279G were prepared as described.^7^ BC2T‐FB used for structure determination and analytical SEC analysis also carried the S699A mutation to render it inactive. The two‐chain monomers FPc, FPΔ3, and FPΔ2,3 were prepared as described.^7^, ^15^ CVF was purified from lyophilized Naja naja siamensis venom that was dissolved at a concentration of 50 mg/ml in a buffer containing 50 mM HEPES pH 7.5, 150 mM NaCl for 12 hr at room temperature with gentle shaking. The resuspended venom was filtered through a 0.22 μm filter prior to SEC on a 120 ml Superdex 200 PG column (GE Healthcare) equilibrated in the resuspension buffer. Fractions containing CVF based on SDS‐PAGE analysis were pooled.

### 4.2 Preparation of nanobodies

His_6_‐tagged bispecific fusion nanobody BC2‐hFPNb1 was expressed in the *Escherichia coli* BL21(D3). Cells harboring the plasmid were grown at 37°C in 2× yeast tryptone medium. At OD_600_ = 0.8, the cells were incubated on ice for 30 min followed by 30 min incubation at 18°C, before protein expression was induced with 0.5 mM isopropyl β‐d‐1‐thiogalactopyranoside, and growth was continued at 18°C overnight. All purification steps were performed at 4°C. The cells pellets were resuspended in 1x PBS pH 7.5, 400 mM NaCl, 20 mM imidazole, and 1 mM PMSF. The cells were lysed by sonication, and the cell debris was removed by centrifugation. The supernatant was loaded on a 1 ml HisTrap FF Crude column (GE Healthcare). The column was washed in 15 ml of 1x PBS pH 7.5, 400 mM NaCl, 20 mM imidazole, and the protein was eluted in the same buffer with 400 mM imidazole. To prepare BC2‐hFPNb1 for crystallization, the eluate was added 1:5 w:w TEV protease to remove the His_6_‐tag and dialyzed against 20 mM HEPES pH 7.5, 300 mM NaCl overnight. After complete TEV cleavage, a negative purification was performed on a 1 ml HisTrap FF Crude column as described above. The flow through containing the untagged BC2‐hFPNb1 was dialyzed into 50 mM Tris pH 8, 25 mM NaCl overnight. Subsequently, BC2‐hFPNb1 was applied to a 1 ml Mono Q column (GE Healthcare) pre‐equilibrated in 50 mM Tris pH 8, 20 mM NaCl, and eluted by a 20 ml linear gradient from 20 to 250 mM NaCl. A final polishing step was performed by SEC on a 24 ml Superdex 75 column (GE Healthcare) equilibrated in 20 mM HEPES pH 7.5, 150 mM NaCl. To prepare BC2‐hFPNb1 for BLI, the protein was purified as described below for the BC2 nanobody.

The BC2 nanobody was expressed in the *E. coli* BL21(D3) and purified by affinity chromatography on a 1 ml HisTrap FF Crude column as described above. The eluate was dialyzed into 2 × 2 L of 20 mM Na‐acetate pH 5.5, 50 mM NaCl, before the BC2 nanobody was applied to a 1 ml Source 15S column (GE Healthcare) pre‐equilibrated in 20 mM Na‐acetate pH 5.5, 50 mM NaCl. The BC2 nanobody was eluted by a 20 ml linear gradient from 150 to 400 mM NaCl. The fractions containing BC2 were pooled and concentrated, before a final SEC step on a 24 ml Superdex75 column (GE Healthcare) pre‐equilibrated in 20 mM HEPES pH 7.5, 150 mM NaCl. The hFPNb1 was purified as described in Reference 34.

### 4.3 Analytic SEC analysis of complex assembly

Analytical SEC involving C3b, BC2T‐FB D279G‐S699A, FPc, and BC2‐hFPNb1 was performed on a 24 ml Superdex 200 increase column (GE Healthcare) equilibrated in 20 mM HEPES pH 7.5, 150 mM NaCl. For the BC2T‐FB:FPc:BC2‐hFPNb1 complex, BC2T‐FB, FPc, and BC2‐hFPNb1 were mixed in a molar ratio of 1.6:1.6:1 prior to analytical SEC. For the C3b:BC2T‐FB:FPc:BC2‐hFPNb1 complex, C3b was mixed with BC2T‐FB, FPc, and BC2‐hFPNb1 in a molar ratio of 1:1.6:1.6:1. The complex was added 5 mM MgCl_2_ and loaded on the Superdex 200 increase column pre‐equilibrated in 20 mM HEPES pH 7.5, 150 mM NaCl, 5 mM MgCl_2_.

### 4.4 In vitro FD cleavage assay of C3bB

C3b and FB or BC2T‐FB were mixed in a 1.25:1 molar ratio in 20 mM HEPES pH 7.3, 150 mM NaCl, 5 mM MgCl_2_. The mixture was incubated on ice for 5 min before addition of FD (Complement Technology) in a molar ratio of 1:200 to FB/BC2T‐FB. The reaction mixture was incubated on ice, while samples for SDS‐PAGE analysis were taken after 0, 5, 10, 15, 30, 45, 60, 90, and 120 min.

### 4.5 Biolayer interferometry

The BLI experiments were performed on an Octet Red96 (ForteBio) operated at 30°C with shaking at 1,000 rpm. The stability of C3b:BC2T‐FB:FPΔ3 complex with or without bispecific BC2‐hFPNb1 was measured using streptavidin coated biosensors (ForteBio). The running and wash buffer contained 20 mM MES pH 6.0, 100 mM NaCl, 5 mM MgCl_2_, 1 mg/ml BSA, 0.05% Tween20. Biotinylated C3b at 2 μg/ml was loaded on sensors for 300 s and washed for 60 s before loaded with BC2T‐FB at 10 μg/ml for 300 s. Sensors were then washed for 10 s before being transferred to wells with FPΔ3 + BC2 + hFPNb1 (molar ratio 1:1:1) or to wells with FPΔ3 + BC2‐hFPNb1 (molar ratio 1:1). Association and dissociation was monitored for 200 s. The apparent binding constants for the interaction between C3b:FB and FPΔ3 with associated nanobodies were determined by subtracting the C3b:BC2T‐FB curve and fitting the data to a 1:1 Langmuir binding model.

For assessing binding of BC2‐hFPNb1 to BC2T‐FB, the running and wash buffer contained 20 mM HEPES pH 7.5, 150 mM NaCl, 1 mg/ml BSA, 0.05% Tween20. First, anti‐penta‐HIS sensors (ForteBio) were washed for 60 s, followed by a 5 min step where 10 μg/ml of BC2‐hFPNb1 was loaded onto the sensors. Subsequently, the sensors were washed for 60 s and then baselined for 60 s, before the association to BC2T‐FB (220, 110, 55, 27.5, 13.75, 6.87, and 0 nM) was allowed for 5 min, followed by a 5‐min dissociation step. The 0 nM measurements were subtracted from all data series before fitting to a 1:1 Langmuir binding model. The association was modeled as: *R*(*t*) = *R*_max_([BC2T‐FB]/([BC2T‐FB]) + *K*_D_)(1‐exp(−*t*·(*k*_on_·[BC2T‐FB]‐*k*_off_))), where *K*_D_ = *k*_on_/*k*_off_. The dissociation phase starting at 300 s was modeled as a first‐order exponential decay, *R*(*t*) = *R*(300)·exp(−*k*_off_(*t*‐300)).

### 4.6 In vitro C3 cleavage by CVF:Bb and C3b:Bb

To test the activity of BC2T‐FB D279G, a C3 cleavage assay was performed with CVF:Bb and the AP convertase C3b:Bb. The assay was performed in 20 mM HEPES pH 7.5, 150 mM NaCl, 5 mM MgCl_2_. For the CVF:Bb digestion of C3, CVF, BC2T‐FB (or FB), and FD were mixed in the molar ratio of 1.2:1:0.1 and incubated for 15 min at 37°C to allow FD activation of the CVFB complex generating the active convertase CVF:Bb. After activation, the convertase was added purified rat C3 in a 1:10 molar ratio relative to FB and incubated at 37°C for 30 min. For generation of the C3b:Bb convertase, C3b, BC2T‐FB (or FB), and FD were mixed in the molar ratio 0.2:1:0.1, and incubated for 10 min at 37°C. After convertase assembly, human C3 was added in a 1:10 ratio resulting in 1:4 dilution of C3b:Bb, and incubated at 37°C for 30 min. Following digestion with the two convertases, SDS‐PAGE analysis was conducted.

### 4.7 Structure determination

The quaternary complex was assembled by mixing C3b, BC2T‐FB D279G‐S699A, FP∆3, and BC2‐hFPNb1 in the molar ratio of 1:1.6:1.5:1 in gel filtration buffer approximately 5 min before injection onto a 24 ml Superdex 200 increase (GE Healthcare) equilibrated in 20 mM MES pH 6.0, 75 mM NaCl, 1 mM NiCl_2_. Single crystals of the quaternary complex C3b:BC2T‐FB:FPΔ3:BC2‐hFPNb1 were grown by vapor diffusion at 292 K from drops consisting of 150 nl protein solution concentrated to 5 mg/ml and 150 nl reservoir equilibrated against a reservoir of 0.05 M Na‐acetate pH 5.3, 0.1 M Mg‐formate and 7% w/v PEG5000 MME. The crystals were soaked in mother liquor with 20% w/v ethylene glycol added prior to flash freezing in liquid nitrogen. Data were collected at the MAX IV beamline BioMAX at 100 K and processed with XDS^35^ followed by molecular replacement with phenix.phaser.^36^ The resulting model was refined with a rigid body refinement in phenix.refine.^37^ The structure was manually rebuilt in Coot^38^ and refined with phenix.refine in an iterative manner using positional refinement, individual B‐factors and Translation–Libration–Screw (TLS) groups. Structure analysis and figure preparation was done with PyMol (www.pymol.org). Intermolecular interactions were analyzed with PISA.^17^ Notice that Ni^2+^ was used to assemble the complex in large scale, since substitution of the MIDAS Mg^2+^ ion with Ni^2+^ is a common mean of increasing the stability of metal ion‐dependent ligand–vWA domain interactions.^39^ Since 100 mM Mg^2+^ was present in the crystallization reservoir solution and cryo‐protecting solution, the complex was modeled with a Mg^2+^ ion in the MIDAS.

## AUTHOR CONTRIBUTIONS

**Josefine Lorentzen:** Data curation (lead); formal analysis (lead); investigation (lead); methodology (lead); visualization (equal); writing – original draft (equal); writing – review and editing (equal). **Dennis Vestergaard Pedersen:** Conceptualization (equal); data curation (equal); formal analysis (equal); investigation (equal); methodology (equal); supervision (lead); validation (equal); visualization (equal); writing – original draft (equal). **Trine Amalie Fogh Gadeberg:** Investigation (equal); methodology (equal); writing – review and editing (equal). **Gregers Rom Andersen:** Conceptualization (lead); data curation (equal); formal analysis (equal); funding acquisition (lead); methodology (supporting); project administration (lead); resources (lead); supervision (lead); validation (equal); visualization (equal); writing – original draft (lead); writing – review and editing (lead).

## CONFLICT OF INTEREST

The authors declare no potential conflict of interest.

## Supporting information


**APPENDIX S1** Supporting Information

## Data Availability

Structure factors and coordinates for the structure of the C3b‐FB‐FP complex are available in the protein data bank as entry 7NOZ.
