# Supplementary material for: Structure determination of an unstable macromolecular complex enabled by nanobody‐peptide bridging
Source: Protein Sci. 2022 Sep 27;31(10):e4432. doi: 10.1002/pro.4432 (PMC9601772; doi:10.1002/pro.4432)
Supplement: Supplementary file 1 — APPENDIX S1 Supporting Information [file PRO-31-e4432-s001.docx]

**
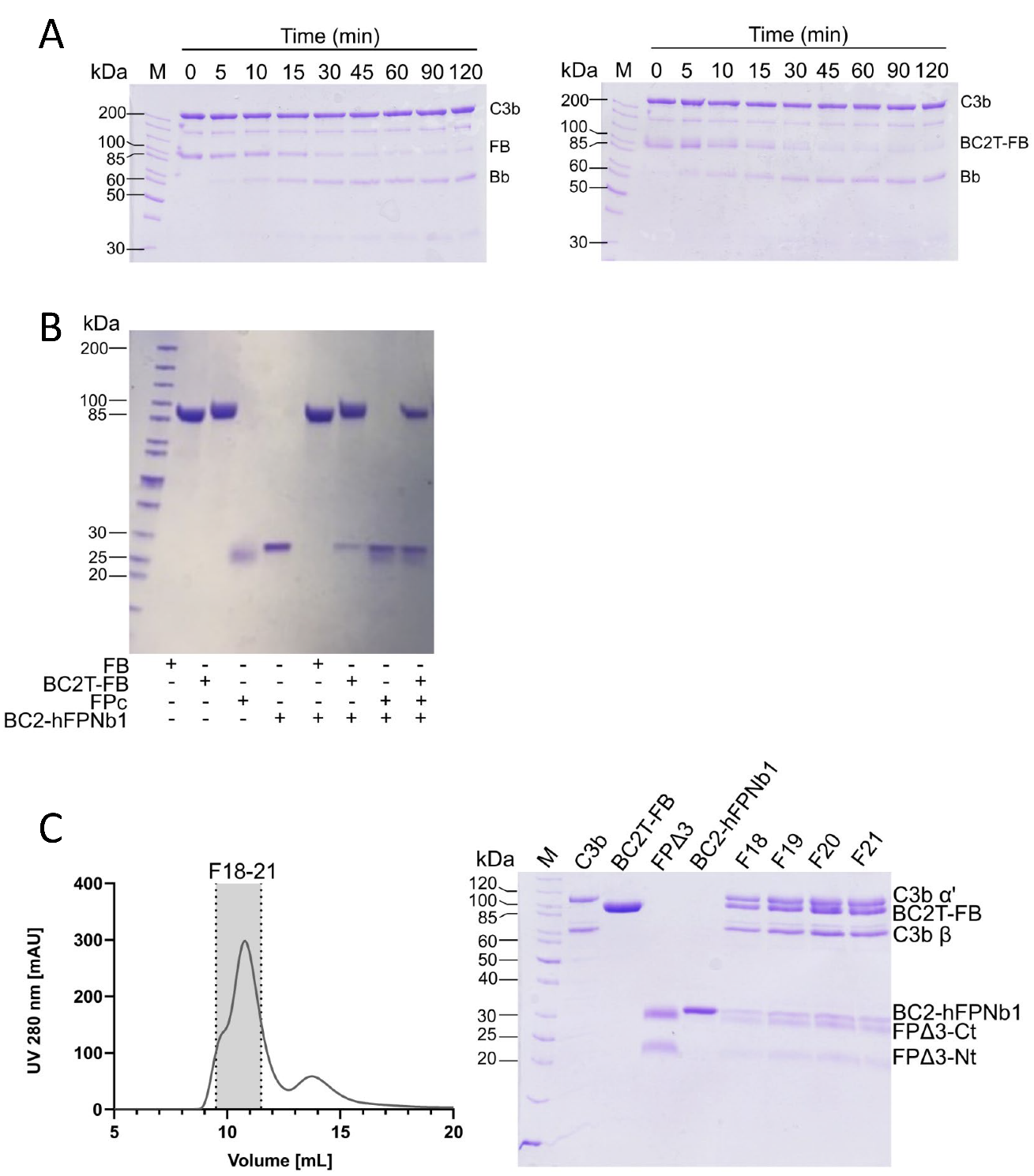
**

**Figure S1. Analyses of BC2T-FB functionality and formation of C3b:BC2T-FB:FP:BC2-hFPNb1 complexes.** A) SDS-PAGE analysis of the FD cleavage of FB (left) and BC2T-FB (right) to assess the functionality of BC2T-FB. Similar cleavage rates are observed for the two FB variants. B) SDS-PAGE analysis of the first peak from eight separate SEC runs of the components used for the experiment presented in figure 2D. Notice that the two chains in FPc have the same mobility. C) Elution profile of large scale complex formation of C3b:BC2T-FB:FPΔ3:BC2-hFPNb1 and reducing SDS-PAGE analysis of the peak fractions used for crystallization. In this experiment, the amounts used of the individual components were 550 µg C3b, 470 µg BC2T-FB, 220 µg FPΔ3, 100 µg BC2-hFPNb1. The early fractions possibly contain dimers of the complex, but the ratio between the subunits appears to be conserved in the analyzed fractions.

**
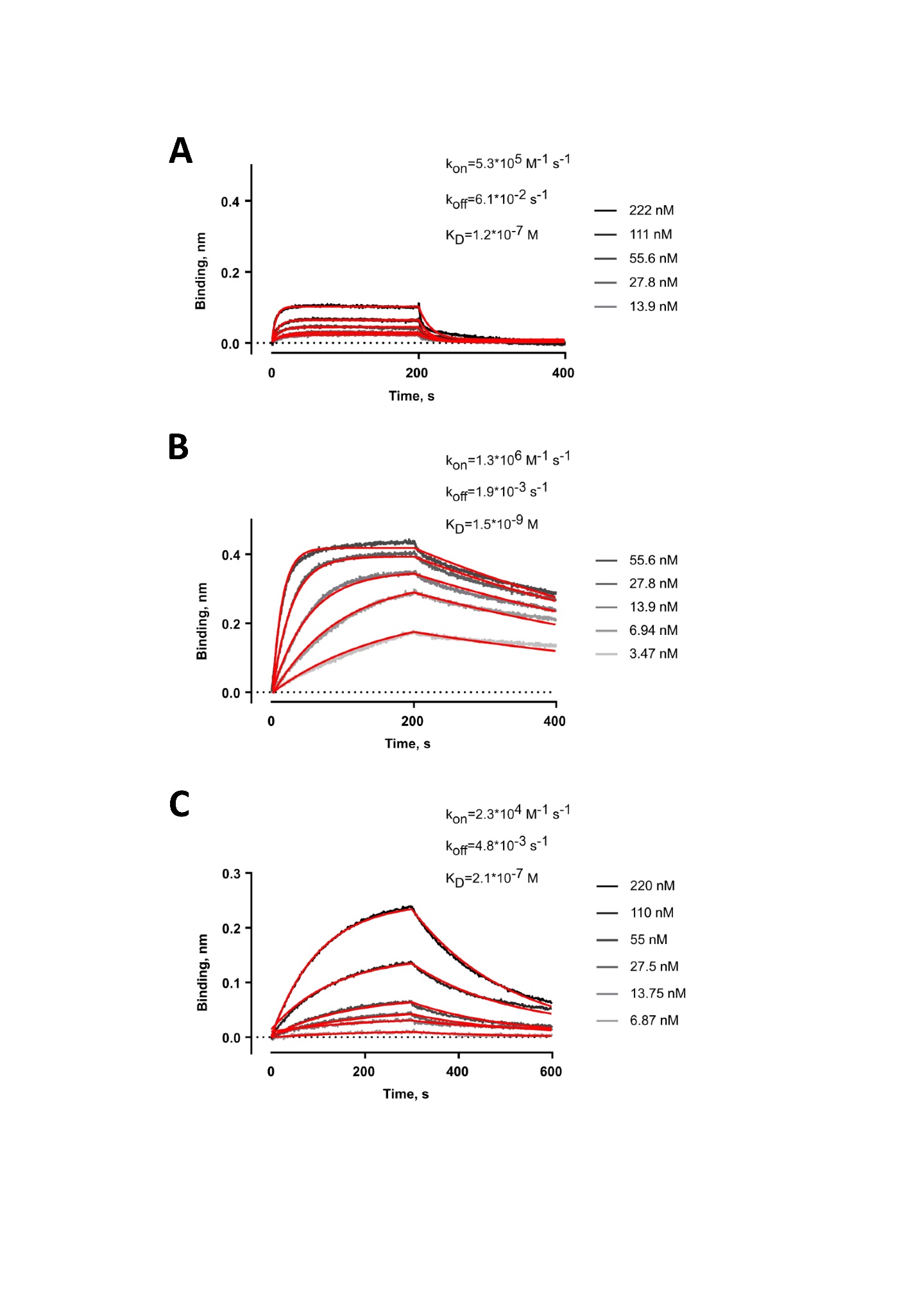
**

**Figure S2. BLI analysis demonstrates the high stability of the nanobody bridged C3b:BC2T-FB:FPΔ3 complex.** In grey the experimental binding curves and in red the fitted curves. Rate constants and dissociation constant calculated from BLI sensorgrams are presented at each. A-B) The experimental curves (shades of grey) obtained from the right parts of Figure 2E-F are displayed together with fitted curves (red) obtained by using a 1:1 Langmuir binding model to derive apparent values for k_on_, k_off_ and K_D_. Due to the slow dissociation of BC2T-FB, these curves are assumed to mainly represent FPΔ3 dissociation from the C3b:BC2T-FB complex at least in panel A. C) BLI analysis of the interaction between immobilized BC2-hFPNb1 and BC2T-FB present in the wells at the indicated concentrations.

MQVQLVESGGGLVQPGGSLTLSCTASGFTLDHYDIGWFRQAPGKEREGVSCINNSDDDTYYADSVKGRFTIFMNNAKDTVYLQMNSLKPEDTAIYYCAEARGCKRGRYEYDFWGQGTQVTVSS**GGGGSGGGGSGGGGS**MQVQLVESGGGLVQAGGSLRLSCAASERTFTIYAMGWFRQAPGKEREFVAAISRSGENTDYADSVKGRFTISRDNNKNTISLQMNSLKPEDTAVYYCAAGRAILVHTTKKEYDHWGQGTQVTVSSENLYFQ

TPG**PDRKAAVSHWQQ**TPWSLARPQGSCSLEGVEIKGGSFRLLQEGQALEYVCPSGFYPYPVQTRTCRSTGSWSTLKTQDQKTVRKAECRAIHCPRPHDFENGEYWPRSPYYNVSDEISFHCYDGYTLRGSANRTCQVNGRWSGQTAICDNGAGYCSNPGIPIGTRKVGSQYRLEDSVTYHCSRGLTLRGSQRRTCQEGGSWSGTEPSCQDSFMYDTPQEVAEAFLSSLTETIEGVDAEDGHGPGEQQKRKIVLDPSGSMNIYLVLDGSGSIGASDFTGAKKCLVNLIEKVASYGVKPRYGLVTYATYPKIWVKVSEADSSNADWVTKQLNEINYEDHKLKSGTNTKKALQAVYSMMSWPDDVPPEGWNRTRHVIILMTDGLHNMGGDPITVIDEIRDLLYIGKDRKNPREDYLDVYVFGVGPLVNQVNINALASKKDNEQHVFKVKDMENLEDVFYQMIDESQSLSLCGMVWEHRKGTDYHKQPWQAKISVIRPSKGHESCMGAVVSEYFVLTAAHCFTVDDKEHSIKVSVGGEKRDLEIEVVLFHPNYNINGKKEAGIPEFYDYDVALIKLKNKLKYGQTIRPICLPCTEGTTRALRLPPTTTCQQQKEELLPAQDIKALFVSEEEKKLTRKEVYIKNGDKKGSCERDAQYAPGYDKVKDISEVVTPRFLCTGGVSPYADPNTCRGDAGGPLIVHKRSRFIQVGVISWGVVDVCKNQKRQKQVPAHARDFHINLFQVLPWLKEKLQDEDLGFL

**Table S1. Sequence of proteins developed for the study**. Top, the BC2-hFPNb1 fusion protein after TEV cleavage with (GGGGS)_3_ linker in bold. Bottom, the BC2T tagged FB with the BC2 tag in bold (bottom).
